# Utility of Ultrasound and Mammography in Detection of Negative Axillary Nodal Metastasis in Breast Cancer

**DOI:** 10.7759/cureus.6691

**Published:** 2020-01-17

**Authors:** Anam Khan, Imrana Masroor, Kumail Khandwala, Summar-un-nisa Abbasi, Muhammad Usman Tariq

**Affiliations:** 1 Radiology, Aga Khan University Hospital, Karachi, PAK; 2 Pathology, Aga Khan University Hospital, Karachi, PAK

**Keywords:** ultrasound, mammography, sentinel lymph node, biopsy, histopathology, metastasis, lymphadenopathy

## Abstract

Objective

The status of axillary lymph nodes is one of the most important prognostic factors in patients with breast cancer. A precise noninvasive evaluation of axillary lymph node status preoperatively, although challenging, is vital for optimization of the treatment plan for patients. The objective of our study was to assess the utility of ultrasound and mammography in detecting the absence of axillary lymph nodal metastasis in patients of breast cancer, taking histopathology as gold standard.

Methods

A cross-sectional study was conducted in the Department of Radiology, Aga Khan University Hospital, Karachi. All female patients between 20 and 95 years of age with a known diagnosis of breast cancer with mammographic and ultrasound imaging done at our institute were included. Patients with abnormal lymph nodes on mammography or on ultrasound, patients already operated for breast cancer, patients who already underwent axillary lymph node dissection and those whose histopathology reports were not available or who did not undergo surgery were excluded.

Results

A total of 262 women with breast carcinoma who had both ultrasound and mammography done and also had surgery performed at our institution were included. At final surgical pathology, a total of 45 of the 262 patients (17.2%) with breast carcinoma had one or more positive lymph nodes. Out of the total 262 patients, 217 patients were found to be true negatives as they had absent axillary nodal metastasis on imaging as well as on histopathology. In all, 45 out of 262 patients were found to be false negatives as they had absent axillary nodal metastasis on imaging; however, they were found to be positive for metastasis on histopathology. The negative predictive value was 82.8%. Patient age was considered as a factor that may influence the outcome of results; the patients were stratified into age ranges seven groups with the age range of 10 years, ranging from 26 to 95 years. Chi-square test showed a *p*-value of 0.148, which showed no significant difference in the effect of age on diagnosing the absence of metastasis by ultrasound and mammography.

Conclusion

Our study shows that ultrasound and mammography even when used in combination cannot safely exclude axillary metastasis and thus cannot eliminate the need for sentinel node biopsy.

## Introduction

Primary breast cancer is one of the most commonest malignancies in females worldwide [1]. Pakistan has the highest incidence rate in Asia, with approximately one in every nine women suffering from breast cancer [2]. The Karachi cancer registry reported breast cancer as the most common cancer (34.6%) among females in Pakistan with an estimated incidence rate of 50 in 100,000 [2-3]. The mortality and morbidity associated with high disease burden of breast cancer can be decreased by early detection of breast cancer.

Breast imaging plays an essential role in the diagnosis and management of breast disease, using a multimodality approach, including X-rays, ultrasound, magnetic resonance imaging, and nuclear medicine techniques [4]. Axillary lymph node status remains the most important breast cancer prognostic factor and is essential for establishing treatment decisions. The standard for determining axillary involvement is sentinel lymph node biopsy (SLNB). SNLB uses a radiotracer to identify the first node or nodes draining breast and thus the initial nodes to encounter metastatic disease. It is usually performed at the time of surgical resection and has an accuracy of 93.5% to 97.5% [5-8]. This invasive surgical procedure carries associated morbidity including longer surgical time, an additional surgical scar, painful preoperative injections, lymphedema, seroma, and possible sensory paresthesias [9,10].

Currently, no other noninvasive alternative diagnostic technique as accurate as the sentinel lymph node technique for staging the axillary lymph nodes is known. Numerous nonsurgical diagnostic methods including physical examination, mammography, ultrasound, computed tomographic scan, magnetic resonance imaging, and positron emission tomographic imaging have been used with variable success to detect lymph node involvement [11-20].

Mammography is the standard imaging modality used in screening for breast disease. The accuracy of mammography has been previously reported to be 79.5% with a sensitivity of 21%. Axillary ultrasound is routinely used preoperatively to evaluate the involvement of lymph nodes. Ultrasound has accuracy of 82.8%, sensitivity 21%, and specificity 99.5% [21]. However, accurate screening to determine whether axillary lymph nodes are involved in the metastatic process and subsequent needle biopsy is required remains a challenge.

The objective of this study was to assess the utility of the combination of ultrasound and mammography, taking histopathology as the gold standard, to detect negative axillary lymphadenopathy in breast carcinoma. Therefore, we aim to increase the confidence in declaring the axilla negative for metastatic involvement of nodes, thus potentially eliminating the need for SNLB and saving the patient from additional pain, cost and most importantly radiation exposure. This is the first study from the developing country of Pakistan to encourage the widespread and appropriate use of a combination of different diagnostic modalities for ruling out metastatic involvement of axillary lymph nodes in breast cancer patients.

## Materials and methods

This was a retrospective cross-sectional study conducted at the Department of Radiology, Aga Khan University Hospital, Karachi. This study was conducted from July 26, 2016 to June 26, 2018 for a duration of 23 months. We included female patients between 20 and 95 years of age with a known diagnosis of breast cancer who were referred to us from the breast surgery consulting clinics. Patients with abnormal lymph nodes on mammography or on ultrasound, patients already operated for breast cancer, patients who already underwent axillary lymph node dissection, and those whose histopathology reports were not available or who did not undergo surgery were excluded.

Mammogram consisted of mediolateral oblique and craniocaudal views with any additional views, if required. Mammograms were evaluated for any abnormal appearing lymph nodes by board-certified attending radiologists with experience in women imaging of at least five years. Lymph nodes were considered abnormal on mammogram if the size was more than 2 cm (in short axis), or if they were irregular or rounded in shape, with spiculated margins, absence of lucent appearing fat within the lymph node representing the loss of fatty hilum, or if the density was increased [12].

All patients had an axillary ultrasound performed on the side of the involved breast with a high-frequency transducer and any abnormal lymph nodes were recorded. Lymph nodes were documented as abnormal on ultrasound if rounded in shape, long-to-short axis ratio of less than two, appearing more hypoechoic than the surroundings, compression or disappearance of the bright appearing fat within the lymph node, or asymmetry or thickening of the cortex [14-15].

Patients who were found to have negative lymph node status on both the diagnostic modalities (mammography and ultrasound) underwent a sentinel lymph node lymphoscintigraphy, as this is the currently practiced standard for determining axillary involvement. 37 MBq of radiolabelled 99m-Technetium was injected in the periareolar region. The patients were scanned on GE dual-head gamma camera at 15 minutes, one hour and 24 hours depending on whether the sentinel node was seen or not seen on the gamma camera. The hot node was marked on the skin and operated on the next day under the guidance of a gamma probe. The sentinel node was evaluated for histopathology by frozen section. All lymph node specimens were reviewed by an experienced pathologist for pleomorphism to detect metastatic involvement. The lymph nodes with and without evidence of metastatic involvement were recorded.

Statistical analysis was done using Statistical Package for Social Sciences (SPSS v.19, IBM Corp. in Armonk, NY). Mean and standard deviation were calculated for age. Frequency and percentages were calculated along with true and false positive/negative rates. Effect modifiers were controlled through stratification of age to see the effect of this on outcome variables.

## Results

**Figure 1:**
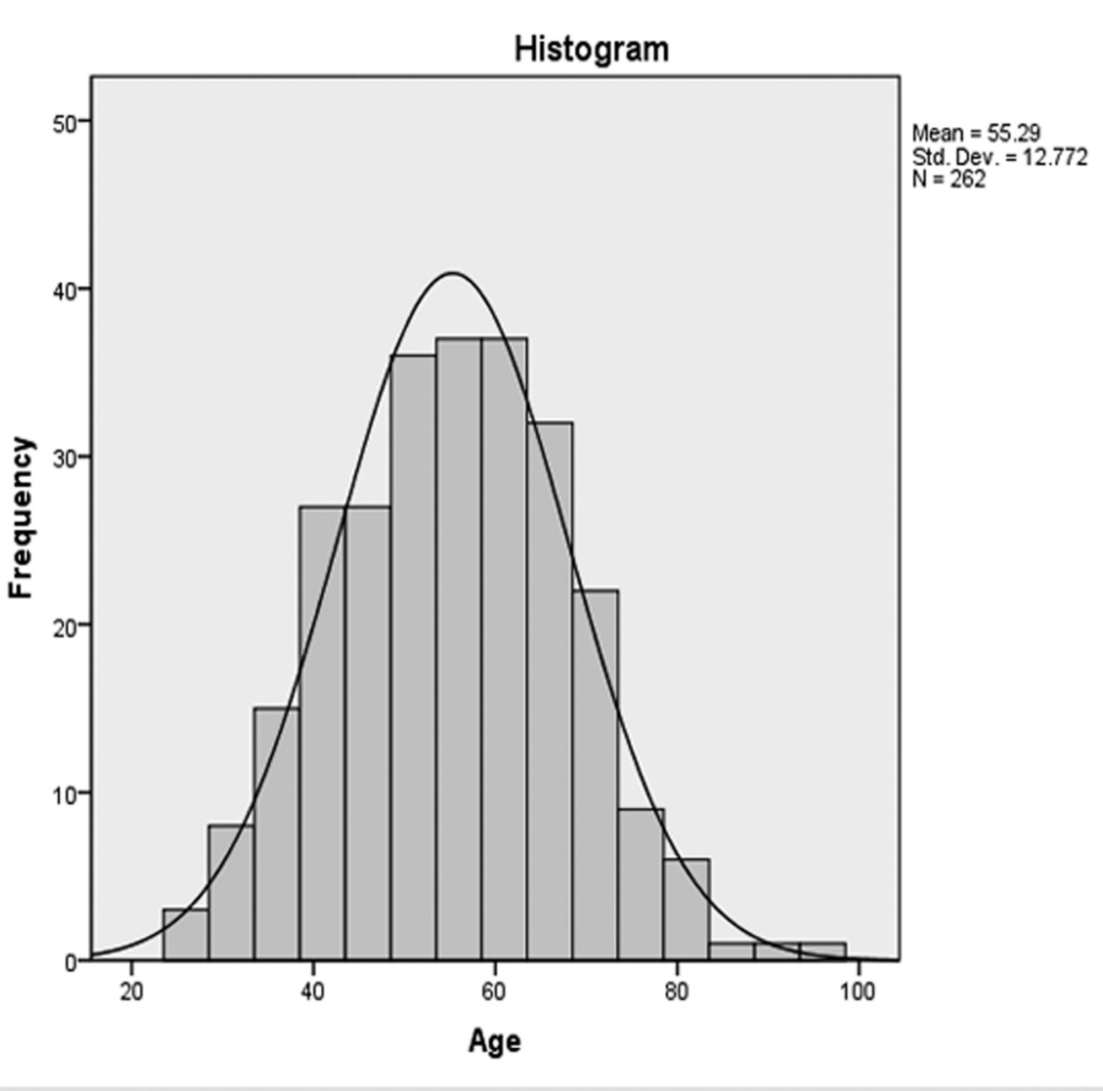
Histogram analysis showing age distributions

We identified and evaluated a total of 262 women with breast carcinoma who had both ultrasound and mammography done and also had surgery performed at our institution. All patients in this study had a mean age of 55.29 years ± 12.78 standard deviation (SD) with an age range of 25 to 95 years (Figure 1).

At final surgical pathology, a total of 45 of the 262 patients (17.2%) with breast carcinoma had one or more positive lymph nodes (Table 1).

**Table 1:** Histopathologic detection of axillary nodal metastasis

Histopathology	Frequency	Percentages (%)
Positive	45	17.2%
Negative	217	82.8%

**Figure 2:**
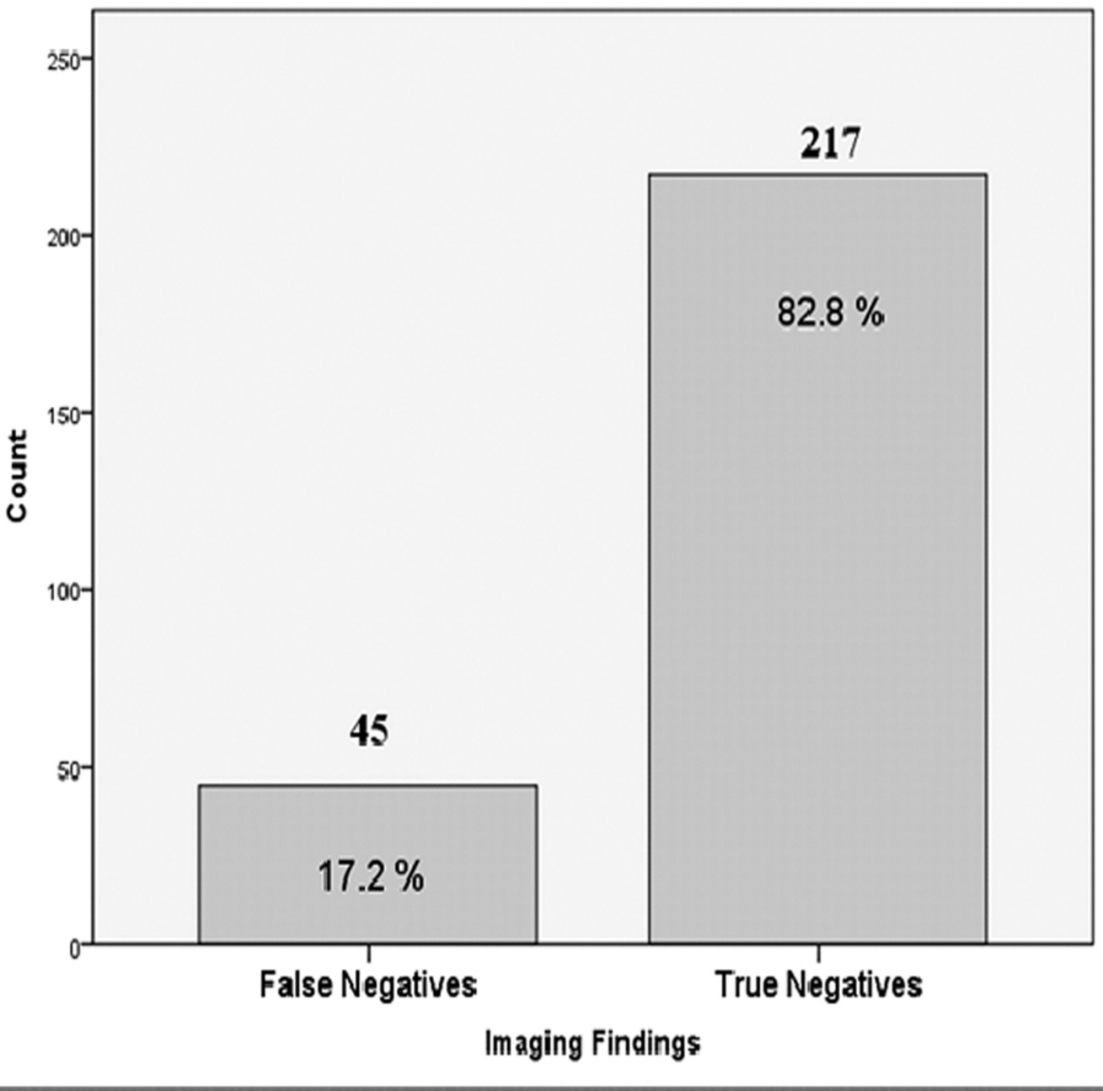
Bar chart showing imaging findings of axillary nodal metastasis

Out of the total 262 patients, 217 patients were found to be true negatives as they had absent metastasis on imaging (ultrasound and mammography) as well as on histopathology. In all, 45 out of 262 patients were found to be false negatives as they had absent metastasis on imaging; however, they were found to be positive for metastasis on histopathology (Figure 2).

The negative predictive value was calculated to be 82.8%. Patient age was considered as a factor that may influence the outcome of results; the patients were stratified into seven groups with age range of 10 years each, ranging from 26 to 95 years (Table 2).

**Table 2:** Post-stratification histopathology data based on age groups Pearson chi-square *P*-value 0.148 (not statistically significant in the association of age groups with axillary nodal metastasis)

Age Ranges	Histopathology		Total
	Positive	Negative	
26-35	1	15	16
36-45	10	34	44
46-55	9	66	75
56-65	13	57	70
66-75	7	35	42
76-85	3	9	12
86-95	2	1	3
Total	45	217	262

**Figure 3:**
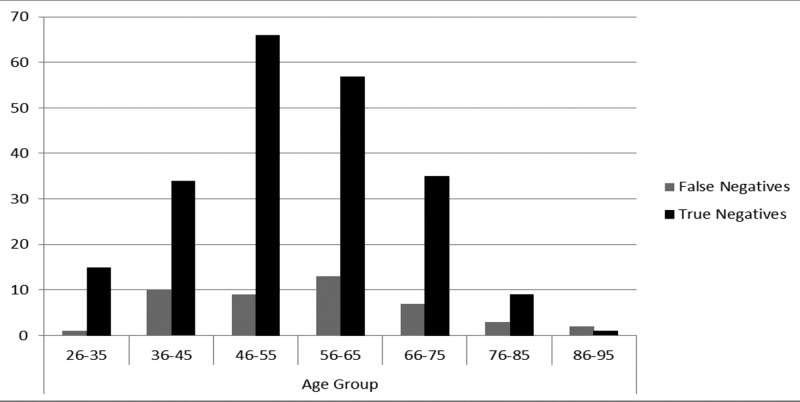
Imaging findings according to age group stratification

There were 16 patients in the age group of 26 to 35 years, histopathology was positive in one of them, 44 patients were present in age range of 36 to 45 histopathology was positive in 10 of them, 75 patients in age group of 46 to 55 years, histopathology was positive in nine of them, 70 patients in age group of 56 to 65 years, histopathology was positive in 13 of them, 42 patients in age group of 66 to 75 years, histopathology was positive in seven of them, 12 patients in age group of 76-85 years, histopathology was positive in three of them and three patients in age group of 86 to 95 years, histopathology was positive in two of them (Figure 3). Chi-square test showed a *p*-value of 0.148 which showed no statistically significant difference among different age groups and the detection of axillary nodal metastasis.

## Discussion

In patients with breast cancer, clinical staging and preoperative planning are of utmost importance because positive axillary lymph node metastasis alters the treatment and surgical options offered to patients. The capability to accumulate this information before surgery has enhanced greatly with the arrival of new imaging and minimally invasive biopsy techniques. Currently, no imaging modality has enough negative predictive value to avoid the need of sentinel node biopsy to the axilla in patients where no lymph node involvement is identified.

Mammography is the standard imaging modality for the screening of breast diseases. Mammography is a less sensitive method for axillary imaging since most of the axilla is pushed out of the image field, and usually, only the lower part is visualized. Ultrasound is a simple test that is used routinely to evaluate lymph node involvement preoperatively.

Our study shows that if both imaging modalities are negative for axillary metastasis, then the patient has 82.8% chance of having negative lymph nodes on final surgical SLNB pathology. These results are comparable to previous similar study which showed that when a combination of physical examination, ultrasound, mammography, and magnetic resonance imaging was negative, the patient had an 86% chance of having negative lymph nodes on final surgical SLNB pathology [21].

**Figure 4:**
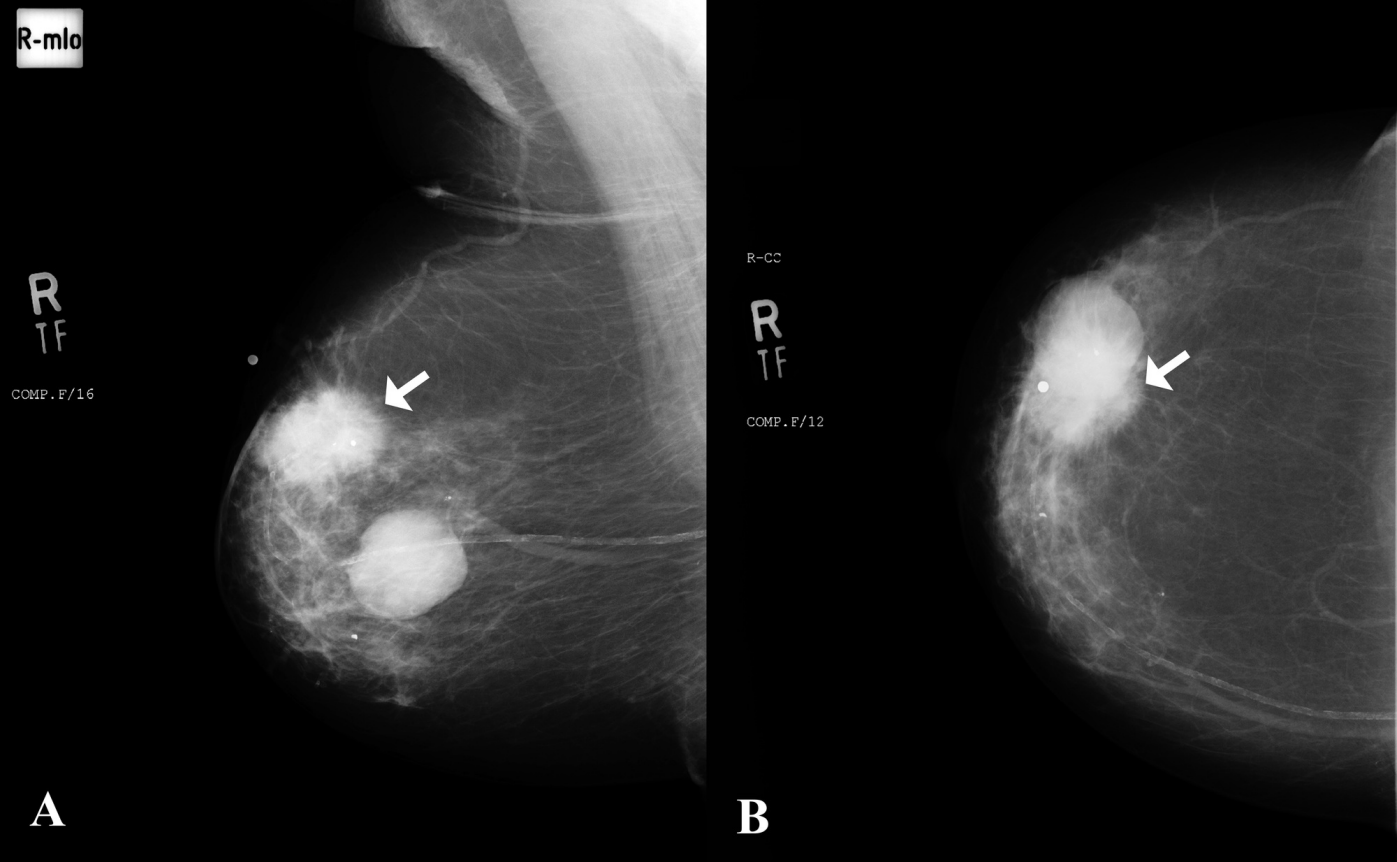
A) Mediolateral oblique and B) craniocaudal views of mammogram in an 82-year-old female Large lesion with spiculated margins in the upper outer quadrant of right breast consistent with a neoplastic lesion (arrows). A well-defined rounded density adjacent to this suspicious lesion was a cyst as correlated on ultrasound. No right axillary lymphadenopathy was noted on mammogram.

**Figure 5:**
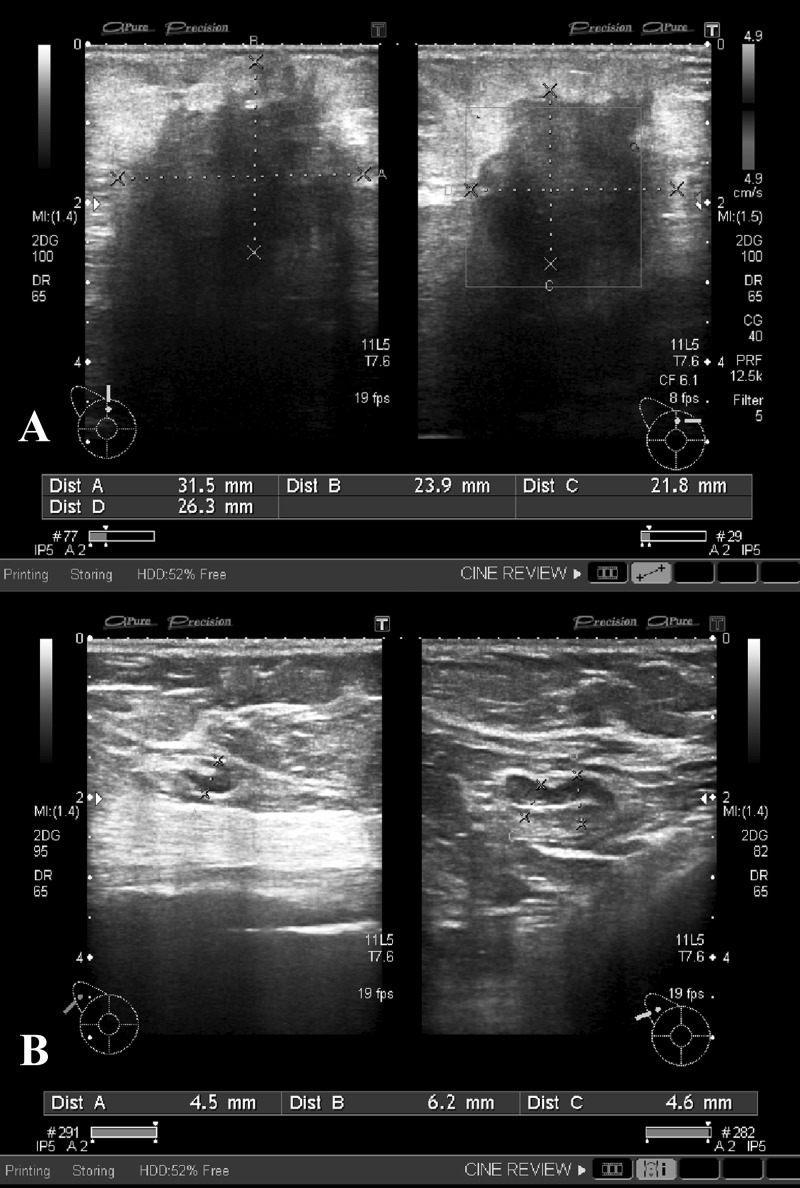
Ultrasound images of right breast and axilla of the patient A) The suspicious neoplastic lesion is redemonstrated in the upper outer quadrant of right breast. B) Subcentimeter lymph nodes with intact fatty hila were identified in the right axilla with thin cortcies and were therefore considered negative for infiltration.

**Figure 6:**
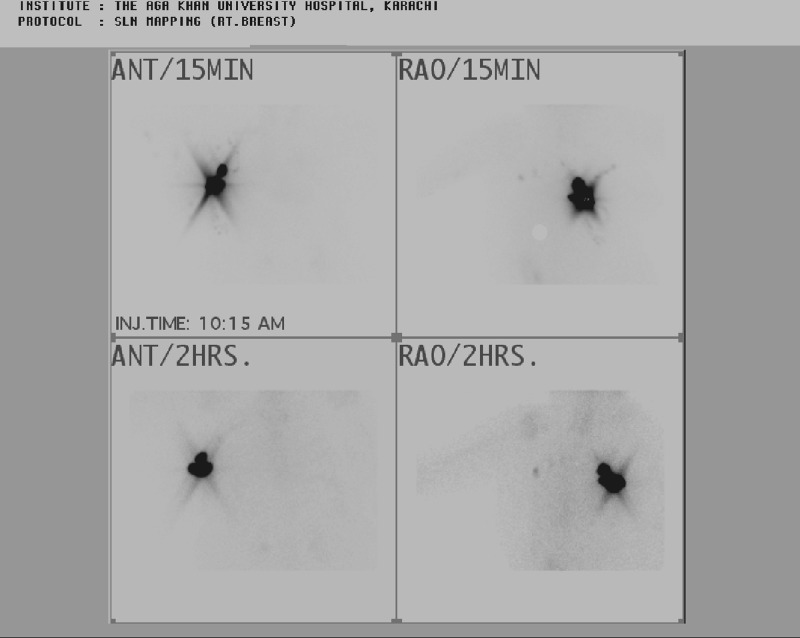
Radionuclide sentinel node mapping and scintigraphy Fifteen-min images show dense tracer uptake over the site of injections and few ill defined areas of abnormal uptake are seen in the right axilla. One hour images redemonstrated dense tracer uptake over the sites of injection and a well outlined and an ill defined area of abnormal tracer uptake in the right axilla. Two nodes were marked over the skin with the help of a hot marker under the gamma camera.

**Figure 7:**
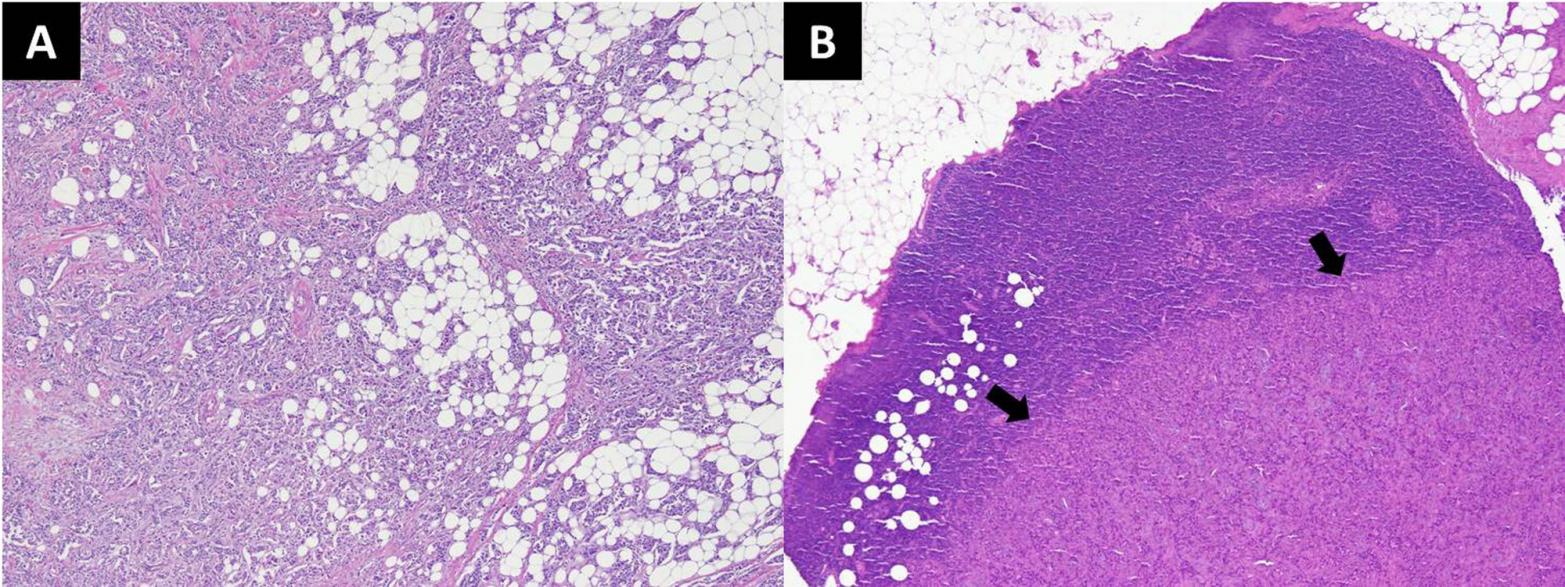
Histopathology slides of the patient A) Invasive ductal carcinoma involving breast tissue. The tumor is arranged in nests, tubules and has infiltrative borders (H&E stain; 40x magnification). B) Metastatic invasive ductal carcinoma involving sentinel lymph node (arrows) (H&E stain; 40x magnification). H&E, hematoxylin and eosin.

False negatives were those who were declared negative for involvement of axillary nodes on imaging but were found to be positive on final surgical histopathology. The false-negative rate of axillary ultrasound and mammography in our study is 17.2%, which is comparable to previously reported 16.7% and 22.9% [21-22]. This means that this still leaves approximately 17% of patients with positive metastasis despite a negative preoperative assessment (Figures 4-7).

For a lymph node with metastatic involvement to be detected on imaging, a macroscopic amount of tumor burden is necessary. Thus, all suspicious lymph nodes cannot be identified by current imaging modalities. Although clinically vital, microscopic diseases cannot be anticipated to be identified. Thus, the negative results on imaging are not reliable because of the high percentage of false negatives. That is to say, a negative ultrasound and mammogram do not exclude lymph node metastasis. The results from our study confirm that presently, ultrasound and mammography in combination cannot consistently identify a subset of patients with a negative assessment in whom sentinel node biopsy may be safely omitted.

One of the limitations of this study was that the cases which were found positive for axillary metastasis on ultrasound or mammography were excluded since in our setup biopsy of the particular positive lymph node is not conducted. Thus, whether that particular lymph node was found positive on final surgical histopathology or not remains indeterminate. Another limitation of our study could be that ultrasound is operator dependent, and lack of perception of an abnormal lymph node by the operator could lead to a false-negative result.

Patient age was considered as a factor that may influence the outcome of results. Age stratification showed the highest number of false-negative patients, 13 out of the total 45 histopathology positive cases, in the age group of 56-65 years; however, these results were statistically insignificant (p-value: 0.148). This is in concordance to the prior study concluding that age is an insignificant factor for determining the absence of axillary metastasis by imaging [23]. Another large population-based study of 13,851 patients carried out by the Danish Breast Cancer Cooperative Group showed that age was a less powerful predictor of the presence of involved nodes [24]. Our results confirm that presently, a combination of ultrasound and mammography cannot be safely used to determine axilla negative for axillary metastasis.

## Conclusions

In conclusion, our study shows that ultrasound and mammography, even when used in combination, cannot safely exclude axillary nodal metastasis and thus cannot eliminate the need of sentinel node biopsy at this point in time.
